# Draft Genome Sequence of mc^2^51, a Highly Hydrogen Peroxide-Resistant *Mycobacterium smegmatis* Mutant Strain

**DOI:** 10.1128/genomeA.00092-14

**Published:** 2014-02-20

**Authors:** Xiaojing Li, Fei Liu, Yongfei Hu, Kaixia Mi

**Affiliations:** a CAS Key Laboratory of Pathogenic Microbiology and Immunology, Institute of Microbiology, CAS, Beijing, China; b University of Chinese Academy of Sciences, Beijing, China; c Beijing Key Laboratory of Microbial Drug Resistance and Resistome, Beijing, China

## Abstract

Here, we report the draft genome sequence of the *Mycobacterium tuberculosis*-like *Mycobacterium smegmatis* mutant strain, mc^2^51, compared to that of wild-type strain *M. smegmatis* mc^2^155. The draft genome sequence comprises 6,823,739 bp, revealing 6,569 coding DNA sequences (CDSs) and 50 RNA genes (4 rRNA genes and 46 tRNA genes).

## GENOME ANNOUNCEMENT

It is known that a successfully established infection of *Mycobacterium tuberculosis* is partially due to its ability to survive and persist in macrophages (1). To defend against infection, the production of reactive oxygen species (ROS) by the host is one of important classical innate mechanisms. Accordingly, to overcome ROS stresses, mycobacteria have evolved many detoxification strategies to scavenge H_2_O_2_, the major contributor of ROS (2). As a model bacterium, *Mycobacterium smegmatis* is widely used for investigating mycobacterial physiology, drug selection, and vaccine development (3–8). Historically, continuous selection was an effective method for making mutant strains. For example, *Mycobacterium bovis* BCG, the only vaccine against tuberculosis, was attenuated by serial passage of *M. bovis in vitro* over 13 years (9). We used a similar adaptive evolution strategy that mimicked the host environment of H_2_O_2_ stress by gradually increasing H_2_O_2_ concentrations to select H_2_O_2_-resistant *M. smegmatis* mutants. After 54-day selection, we obtained the *M. tuberculosis*-like *M. smegmatis* mutant strain, mc^2^51, with an 80-fold higher MIC of H_2_O_2_ and a 100-fold lower MIC (0.1 mg/liter) of isoniazid compared to those of wild-type *M. smegmatis* mc^2^155; this is similar to *M. tuberculosis* with its high resistance to H_2_O_2_ and conversely, susceptibility to isoniazid. In addition, the mutant strain mc^2^51 shows slow growth and has a growth advantage in macrophages compared to wild-type mc^2^155.

We therefore sequenced the whole genome of the mutant strain to explore the genomic variations that are potentially involved into its phenotype. The genome of *M. smemgatis* mc^2^51 was sequenced on an Illumina HiSeq 2000 platform. A total of 4,123,831 paired-end reads with a length of 100 bp, corresponding to 121-fold coverage of the genome, were generated. Raw reads were first filtered using the DynamicTrim and LengthSort Perl scripts provided in the SolexaQA suite (10) and then assembled using SOAP*denovo* (http://soap.genomics.org.cn), yielding 96 scaffolds with an average length of 71,080 bp. The draft genome comprises 6,823,739 bp, with 67.4% G+C content and an N_50_ length of 150,887 bp. The genome was annotated using the Rapid Annotations using Subsystems Technology (RAST) program (11), revealing 6,569 coding DNA sequences (CDSs) and 50 RNA genes (4 rRNA genes and 46 tRNA genes).

We then used the Burrows-Wheeler Aligner (BWA)/SAMtools (12) to call single nucleotide polymorphisms (SNPs). Compared with the parental strain mc^2^155, 22 SNPs were identified in the mutant genome. Nineteen of these mutations are located in the coding regions, and 12 of the 19 SNPs are nonsynonymous mutations. Interestingly, we found nonsynonymous mutations in several genes encoding detoxified enzymes, including dehydrogenase, oxidase, and monooxygenase. We also identified a mutation in the *fur* gene encoding a ferric uptake regulator, which is located immediately upstream of the catalase-peroxidase KatG. The SNPs of *fur* were also found in isoniazid (INH)-resistant clinical isolates, which implies that the mutants of Fur might be produced in humans.

The information will undoubtedly shed light on the ways in which mycobacteria adjust their own fitness and acquire persistent in macrophages, which might give new insights into designing control strategies against *M. tuberculosis.*

### Nucleotide sequence accession number.

The draft genome sequence has been deposited at DDBJ/EMBL/GenBank under the accession no. JAJD00000000. The version described in this paper is the first version.
